# A Pocket Manual for Human Papillomavirus Vaccines

**DOI:** 10.3390/vaccines14030236

**Published:** 2026-03-04

**Authors:** Natalie A. Voss, J. Brooks Jackson, Mary B. Rysavy

**Affiliations:** 1 Department of Pathology, Carver College of Medicine, University of Iowa, Iowa City, IA 52242, USA; natalie-voss@uiowa.edu (N.A.V.); brooks-jackson@uiowa.edu (J.B.J.); 2 Department of Obstetrics, Gynecology, & Reproductive Sciences, McGovern Medical School, University of Texas Health Science Center at Houston, Houston, TX 77030, USA

**Keywords:** HPV vaccination, prevention, safety, efficacy, effectiveness, uptake

## Abstract

Human papillomavirus (HPV) is the most common sexually transmitted infection worldwide and is responsible for the majority of cervical, anal, and vaginal cancers. The first prophylactic HPV vaccine was introduced in the United States in 2006. Extensive evidence demonstrates the HPV vaccine is highly efficacious and effective, particularly when administered prior to HPV exposure. Despite strong safety data and proven cost-effectiveness, HPV vaccine uptake in the United States and globally remains suboptimal. Barriers to vaccination include limited knowledge, safety concerns, and logistical challenges. Current advancements focus on single-dose vaccine regimens, development of therapeutic vaccines, and higher-valent formulations. Expanding HPV vaccine coverage is essential to reduce HPV-related diseases, strengthen herd immunity, and advance cancer prevention efforts.

## 1. Introduction

Human papillomavirus (HPV) is a non-enveloped, double-stranded DNA virus belonging to the Papillomaviridae family. The HPV virus was first described in 1949 by Straus et al. using electron microscopy. In the 1970s, Professor Harald zur Hausen first postulated a causal link between HPV infection and cervical cancer, and over the following decade his laboratory identified HPV 16 and HPV 18 in cervical cancer biopsies [1].

HPV virions are icosahedral in shape, and the viral genome consists of three function regions that encode eight proteins. The long control region (LCR) regulates gene expression and replication, the early (E) region encodes proteins which are required for HPV gene expression, replications and survival, and the late (L) region encodes viral structural proteins [2,3]. The structural proteins include L1 and L2, which form the outer protein capsid [2,4].

The mechanism of HPV infection of cutaneous and mucosal epithelial cells is not fully understood. The most commonly accepted mechanism is that HPV first infects the basal layer of epithelial cells through microtraumas, and once the infected epithelial cells differentiate, the virus replicates to a high copy number and expresses capsid genes (L1 and L2), resulting in new virions that are released from the epithelial surface [3]. The epithelial transition zones, such as the endo-/ectocervix and anorectal junctions, are regions that are more susceptible to carcinogenesis [5].

**Figure 1:**
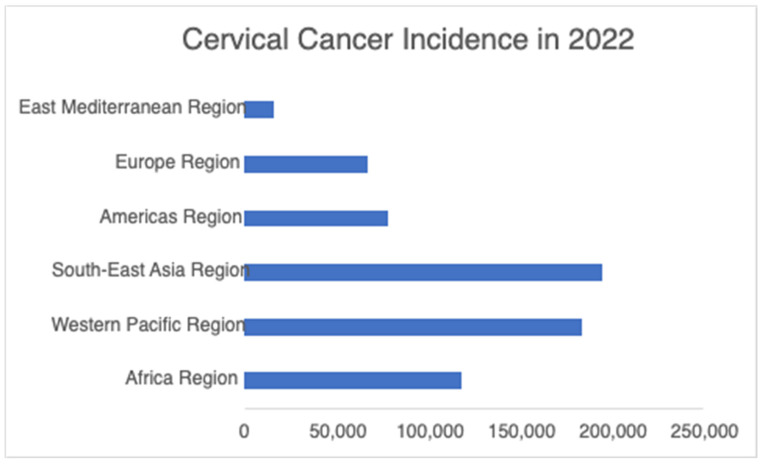
Estimated number of new cervical cancer cases by WHO region in 2022. The highest incidence was in the South-East Asia and Western Pacific regions, followed by Africa, emphasizing persistent geographic disparities in cervical cancer burden. Data derived from global cancer estimates compiled by the International Agency for Research on Cancer [12].

Globally, HPV is the most common sexually transmitted infection. Transmission occurs primarily through sexual contact, including vaginal, anal, and oral sex [6]. Although perinatal transmission during childbirth is possible, vertical transmission rates remain low [7]. Importantly, individuals with HPV can transmit the virus even in the absence of symptoms [6]. The World Health Organization (WHO) estimates that ~80% of sexually active individuals will contract HPV at least once. More than 90% of HPV infections are asymptomatic, do not cause disease, and are cleared within 12–24 months [3,8,9]. However, persistent infection may lead to preneoplastic lesions that may further result in cancer. HPV is estimated to be responsible for approximately 99% of cervical cancers, 90% of anal cancer, 65% of vaginal cancers, and 45–90% of oropharyngeal cancers [8,10]. The cervical cancer burden according to WHO region is summarized in Figure 1 [11]. While HPV infects both men and women, the burden of disease is higher in women due to HPV infection of cervical cells [3].

More than 200 HPV types have been identified [13], of which approximately 14 are considered high-risk because of their potential oncogenicity. The high risk-strains are HPV 16, 18, 31, 33, 35, 39, 45, 51, 52, 56, 58, 59, 66, and 68 [14,15]. HPV-related oncogenesis is primarily mediated by the viral oncoproteins E5, E6 and E7. The oncoproteins E6 and E7 inhibit p53 and pRB tumor suppressors respectively. The oncoprotein E5 assists the virally infected cells with proliferation, inhibition of apoptosis, and immune evasion during the early stages of malignant transformation [3,16,17]. High-risk HPV types 16 and 18 are the cause of ~70% of cervical cancers worldwide. Low-risk HPV types, primarily HPV 6 and HPV 11, account for around 90% of genital warts and the majority of recurrent respiratory papillomatosis lesions [8,18]. In the United States, an estimated 42 million people are infected with disease-causing genital HPV-type, with approximately 13 million new infections occurring annually. HPV is implicated in approximately 39,300 cancers each year in the United states and an estimated 690,000 cancer cases globally [8,19].

## 2. HPV Vaccination

### 2.1. HPV Vaccine Background

In the early 1950s, Henrietta Lacks developed cervical cancer and her cancer cells led to the discovery of the link between HPV and cervical cancer in the 1970–1980s [20,21]. Following the establishment of this association, HPV vaccine development was initiated in Australia in 1991 when Dr. Ian Frazer and Dr. Jian Zhou successfully engineered virus-like particles (VLPs) based on the L1 proteins expressed on the HPV viral capsid. These VLPs form the basis of current HPV vaccines and contain no viral DNA, rendering them noninfectious [22].

The first prophylactic HPV vaccine, Gardasil (Gardasil-4), was licensed in 2006 and targeted HPV types 6, 11, 16, and 18. Extensive clinical trials spanning seven years demonstrated that the vaccine offered nearly 100% protection against the high-risk types HPV 16 and 18 in HPV-naïve individuals; it was approved for use in girls aged 9–26 in the US. In 2009, the US Food and Drug Administration (FDA) expanded approval to include boys. A second vaccine, the bivalent Cervarix, was licensed in 2007 and provided protection against HPV types 16 and 18. In 2014, a third vaccine, Gardasil 9, was licensed; this nonavalent vaccine targets HPV types 6, 11, 16, 18, 31, 33, 45, 52, and 58, thereby expanding coverage against oncogenic HPV strains [2,23]. Additional HPV vaccines have since been developed and are summarized below.

All licensed HPV vaccines, summarized in Table 1 [24], share a common mechanism of action based on virus-like particles (VLPs), which are recombinant, noninfectious assemblies of the HPV L1 capsid protein. The VLPs contain no viral DNA, but they are antigenically indistinguishable from native, infection-causing HPV virions. The VLPs’ administration elicits a robust neutralizing antibody response that prevents viral entry into the basal epithelial cells [18]. The antibody-mediated humoral immunity underlies the high prophylactic efficacy of HPV vaccines. Consistent with this mechanism, vaccine effectiveness is reduced in individuals with prior HPV exposure, as infection of basal epithelial cells have already occurred [25]. Studies on cross-protection against non-vaccine HPV types have been inconsistent, and while it may occur, it has been found to decline faster than vaccine specific protection [26]. Current HPV vaccination strategies are primarily prophylactic and emphasize administration prior to the onset of sexual activity, when individuals are more likely to be HPV-naïve.

### 2.2. Current Vaccine Recommendations

WHO now recommends:

1. A one or two-dose schedule for girls aged 9–14 years
2. A one or two-dose schedule for girls and women aged 15–20 years
3. Two doses with a 6-month interval for women older than 21 years

The primary target of vaccination is girls aged 9–14, prior to the start of sexual activity. The secondary targets such as boys and older females are recommended when feasible and affordable [27].

WHO also highlights the importance of vaccinating immunocompromised people and those living with human immunodeficiency virus (HIV). They recommend these individuals should receive at a minimum two doses and where possible, three doses of the HPV vaccine [27].

Individuals with a history of a life-threatening allergic reaction to a prior dose of the HPV-vaccine or individuals with a yeast allergy should not receive the HPV vaccine. Vaccination should be deferred in individuals with a moderate or severe febrile illness until symptoms have resolved. Because of a risk of fainting and allergic reactions, an observation period of at least 15 min should occur after the vaccine is received [28].

## 3. Efficacy of HPV Vaccines

Human papillomavirus vaccines demonstrate exceptional efficacy in controlled clinical trial settings. HPV vaccines have greater than 99% efficacy when administered to women who have not been exposed to the HPV type of the vaccination [29]. In a randomized controlled trial of females aged 15–26 who were HPV-negative at baseline, HPV vaccines had at least 96% efficacy for preventing cervical precancers (CIN2+), 99% efficacy for high-grade lesions (CIN3+), and 90% efficacy in adenocarcinoma in situ (AIS) caused by vaccine-targeted HPV types [8]. Other trials had similar findings, documenting efficacy between 90% and 100% against vaccine-targeted types for cervical endpoints of persistent infection through CIN3 in HPV-naïve women [30,31].

In males aged 10–26 years, the quadrivalent vaccine demonstrated 90.4% efficacy for preventing vaccine type-related external genital lesions in per-protocol populations, and 100% efficacy for preventing anogenital warts [8]. In a separate randomized trial, intention-to-treat analysis showed a 60.2% reduction in external genital lesions (36 cases in the vaccine group versus 89 in the placebo group), with the efficacy of 65.5% for lesions attributable to HPV types 6, 11, 16, or 18. Per-protocol efficacy against HPV types 6, 11, 16, or 18-related lesions in this trial was similarly high at 90.4% [32].

Efficacy is much lower in intention-to-treat populations that include individuals with prior HPV exposure. In women aged 24–45 years, per-protocol efficacy was 84.7%, but only 41.6% in the intention-to-treat population due to previous exposure of vaccine-type HPV [8]. In males aged 10–26 years, efficacy against persistent infection with vaccine HPV types was found to be similar in per-protocol populations at 85.6%, and in intention-to-treat populations at 47.8% [32]. This highlights that HPV vaccines are more efficacious if given prior to first HPV infection.

### Immune Response to HPV Vaccination

The serological response to HPV vaccination is much stronger (10–10,000× higher) than the response after natural HPV infection [27,33]. Protection against vaccine-type HPV remains durable without evidence of waning. A study looking at anti-HPV 16 and anti-HPV 18 antibody levels for up to 12 years found that levels remained stable and above natural infection-related antibody levels. In the Nordic extension study of the nonavalent vaccine, more than 2000 women were followed for at least 12 years after, and there were no cases of vaccine-type attributable cervical precancers [34]. Similarly, the Costa Rica Vaccine Trial demonstrated 100% efficacy (95% CI 89.2–100.0) against incident HPV 16/18-related CIN2+ at year 11, with cumulative vaccine efficacy of 97.4% (95% CI 88.0–99.6) over the entire 11-year period [35]. Antibody titers, which are substantially higher than those following natural infections, decrease initially but plateau after around two years [8]. Antibody response after receiving the 9v-HPV vaccine was found to be similar in men and women [36].

## 4. Effectiveness of HPV Vaccine

The HPV vaccine has shown substantial effectiveness in preventing HPV infections and HPV-related diseases at the population level. Within 8 years of vaccine introduction, 4vHPV-type prevalence decreased 71% among adolescents aged 14–19 years and 61% among adults aged 20–24 years [37]. Within 12 years of the initiation of the U.S. vaccination program, the prevalence of vaccine-type HPV infection decreased by 88% among adolescents aged 14–19 years and by 81% among adults aged 20–24 years. Among sexually active females aged 14–24 years, vaccine impact on HPV types 6, 11, 16, and 18 prevalence was 85% overall by 2015–2018, with 90% effectiveness among vaccinated females and 74% among unvaccinated females, demonstrating significant herd protection [8,38,39].

Studies have shown high effectiveness against cervical precancer and cancer. A Cochrane review of 225 studies evaluating over 132 million people found that among those vaccinated at or before age 16, there was an 80% reduced risk of cervical cancer (RR 0.20, 95% CI 0.09–0.44), a 74% reduction in CIN3+ incidence (RR 0.26, 95% CI 0.12–0.56), and reductions in CIN2+ of 41% at medium-term and 62% at long-term follow-up [40]. In England’s national program, cervical cancer incidence was reduced by 87% and CIN3 by 97% in cohorts offered vaccination at age 12–13 compared to the unvaccinated cohort. By mid-2020, England’s program had prevented an estimated 687 cervical cancers and 23,192 CIN3 diagnoses [41,42].

Substantial herd effects have been documented globally. A meta-analysis of data from 14 high-income countries showed that in countries with multi-cohort vaccination and high coverage, anogenital wart diagnosis declined by 88% among girls and 86% among boys under age 20 after 5–8 years, compared to only 44% among girls and 1% among boys in countries with single-cohort vaccination or low coverage. Significant decreases in HPV-type infections have been observed among unvaccinated individuals, indicating population-level protection [43]. A U.S. community study with over 80% vaccination coverage showed a 71.6% reduction in bivalent-type HPV and 75.8% reduction in quadrivalent-type HPV among unvaccinated individuals over 17 years [44]. Another study found an 80.9% decline in quadrivalent vaccine-type HPV detection in women who were vaccinated and a 40% decline among women who were unvaccinated from 2006 to 2017 [45].

Effectiveness has extended to other HPV-related diseases as well. A study conducted between 2008 and 2014 reported significant declines in the prevalence of anogenital warts among females 15–19 years (annual percentage change [APC] = −14.1%; *p* < 0.001), 20–24 years (APC = −12.9%; *p* < 0.001), and 25–29 years (APC = −6%; *p* = 0.001). Significant reductions were likewise observed among men aged 20–24 years (APC = −6.5%; *p* = 0.005) [46]. A study looking at national data of cases of juvenile-onset recurrent respiratory papillomatosis (JORRP) found that incidence of JORRP per 100,000 births declined from 2.0 cases in 2004–2005 to 0.5 cases in 2012–2013 (IRR = 0.2, 95% CI = 0.1–0.4); incidence using state-level data declined from 2.9 cases in 2004–2005 to 0.7 cases in 2012–2013 (IRR = 0.2, 95% CI = 0.1–0.4) [47]. A 2025 hospital-based study found that HPV vaccination was associated with a 92% reduced odds ratio for oral/oropharyngeal cancer (OR 0.008, *p* < 0.0001), with the protective effect remaining significant after adjusting for smoking and alcohol use [48]. A 2021 meta-analysis of six studies with 15,240 participants demonstrated that vaccinated individuals were 46% less likely to develop oral vaccine-type HPV infection (RR 0.54, 95% CI 0.32–0.91) and 80% less likely to develop oral HPV 16 infection specifically (RR 0.20, 95% CI 0.09–0.43) [49]. Since HPV 16 causes over 90% of HPV-related oropharyngeal cancer cases, this finding is particularly significant.

## 5. Safety of HPV Vaccine

HPV vaccines have an excellent safety profile supported by extensive pre- and post-licensure data. More than 270 million doses of the HPV vaccine have been distributed globally since 2006, with no data suggesting severe adverse effects or adverse reactions directly linked to vaccination [29]. The vast majority of side effects when receiving the HPV vaccine include headache, fever, and injection site reaction (redness, pain, swelling). A small number of people may have a more serious side effect, such as fainting or allergic reaction, which is a known risk when receiving any vaccination [50].

VAERS is a national spontaneous reporting system that accepts reports from providers and the public in regard to an adverse event after vaccination and is not designed to find a causal relationship between vaccination and adverse events but to identify possible trends that warrant further study. From 1 January 2015, to 31 December 2024, a total of 23,499 adverse events related to GARDASIL 9 were reported to the VAERS database, with 92.5% of those reports being non-serious events. One study found that adverse event reporting rates were 259 per million 9vHPV doses for all reports and 7 per million doses for serious reports. Another phase III clinical trial found adverse events after 9v-HPV vaccination to be less than 0.1% [51,52,53]. In a post-licensure safety assessment of 600,588 doses of HPV4 vaccine that had been administered to females, no statistically increased risks were found for Guillain–Barré syndrome, stroke, venous thromboembolism, appendicitis, seizures, syncope, allergic reactions, and anaphylaxis [54]. The nonavalent and quadrivalent vaccines have similar safety profiles, except for a higher rate of injection site reactions in the 9v-HPV vaccine [55,56]. A comprehensive review of 109 studies including over 2.5 million vaccinated individuals across six countries found no consistent evidence of increased risk for demyelinating syndromes, complex regional pain syndrome, or postural orthostatic tachycardia syndrome [57].

The most recent report addressing the HPV vaccine from the Global Advisory Committee on Vaccine Safety (GACVS) in 2017 looked at high-quality studies from the US, UK, Denmark, Sweden, and Japan and continued to find no causal link between the HPV vaccine and adverse outcomes that were examined in these studies. They also had a systematic review of serious adverse events (SAEs) following administration of the HPV vaccine conducted, with outcomes being all SAEs, medically significant conditions, new onset of chronic diseases, and deaths. In the review, they found no difference in the rates of SAEs between people who had received the HPV vaccine and people who had not received the HPV vaccine in randomized control trials and good-quality cohort studies [58].

## 6. Cost and Global Access

The HPV vaccine has been found to be cost-effective globally, with particularly favorable economic profiles in low- and middle-income counties where cervical cancer burden is highest. A meta-regression analysis of 613 incremental cost-effectiveness ratios (ICERS) from 75 studies across 195 counties found a mean predicted ICER of $4217 per disability-adjusted life year (DALY) averted globally, with ICERS below $800 per DALY averted in 64 countries. The most favorable cost-effectiveness was observed in Sub-Saharan Africa (population-weighted, adjusted mean ICER of $706 per DALY averted across 46 countries) and South Asia (population-weighted, adjusted mean ICER of $489 across five countries) [59]. These findings support introducing and/or expanding HPV vaccines, especially in countries eligible for subsidized vaccines from programs like Gavi, the Vaccine Alliance, and the Pan American Health Organization.

Cost effectiveness varies by vaccine type. Systematic reviews have found that the bivalent vaccine demonstrated mean ICERs of $3804, the quadrivalent vaccine $640, and then generic HPV 16/18 vaccines $358 in Latin American countries [60]. The nonavalent vaccine has been found to be cost-effective when vaccine prices remain competitive, but it may not be cost-effective in all settings compared to earlier bivalent and quadrivalent vaccines [61]. For example, in a study in the Philippines, Cecolin and Cervarix were much more cost-effective than Gardasil (US$173, US$263, and US$1006 per DALY averted, respectively), and Gardasil-9 was not cost-effective under any of the modeled scenarios [62].

Another study called HPV-ADVISE LMIC used a mathematical modelling analysis to examine the optimal use of limited vaccine supplies and best allocation of scarce resources in four low- and middle-income countries (LMIC): India, Vietnam, Uganda, and Nigeria. That model found that two-dose routine vaccination of girls aged 14 years (with or without later switch to age 9 years) and routine vaccination of girls aged 9 years with a 5-year extended interval between doses plus a catch-up at age 14 years were the most efficient strategies. This strategy minimized both the number of doses needed to prevent one case of cervical cancer (number needed to vaccinate (NNV) ranging from 78 to 381) and cost per DALY averted (from $28 to $1406). Notably, vaccinating boys or women aged 18 years or older resulted in much higher NNVs and ICERS in these settings [63]. Vaccine cost is an important component of total program costs in low- and middle-income countries. A systematic review on the cost of HPV vaccine delivery in LMICs found delivery cost ranged from US$0.37 to US$24.07 per dose for financial costs and $1.48 to $48.70 per dose for economic costs. This evidence suggested that HPV vaccine delivery costs varied widely depending on country and stage of implementation [64].

**Figure 2:**
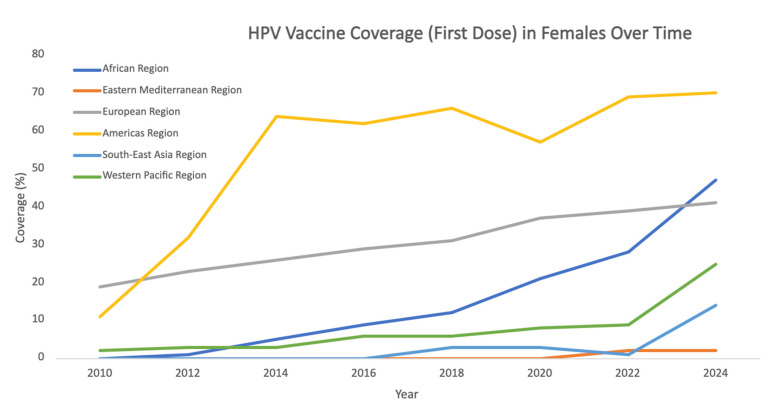
Coverage of first-dose HPV vaccination among females by World Health Organization region since 2010 demonstrates substantial geographic variation. Uptake has increased steadily in several regions, particularly in the Americas and Europe, while progress in Africa and parts of Southeast Asia has been slower, emphasizing inequities in global HPV vaccine implementation. Source: World Health Organization immunization coverage estimates.

Despite demonstrated cost-effectiveness, global HPV vaccination coverage and HPV-related disease burden remains inequitable. Over 80% of cases of cervical cancer occurred in LMICs [63,65]. At the end of 2019, 88% of high-income countries (HICs) had introduced HPV vaccination in girls and women compared to less than 40% of LMICs [63]. In 2022, cervical cancer incidence remained disproportionally high in low- and middle-income regions, with the highest rates observed in Eastern African at 40.4 per 100,000 females, Southern Africa at 34.9, Middle Africa at 28.3, Western Africa at 26.9, and South-Eastern Asia at 17.4. These rates are much higher when compared to high-income regions, where incidence was 4.1 per 100,000 females in Western Asia, 5.2 in Australia-New Zealand, 6.3 in North America, and 6.4 in Southern Europe [66]. Introduction of the HPV vaccine in LMICs was initially prevented by cost, but tiered pricing and lower prices through the Pan American Health Organization’s Revolving Fund (starting in 2011) and Gavi Alliance funding (starting in 2012, with vaccine cost below $5 per dose) has enabled broader access [67]. While vaccine programs have continued to expand and are now in over 150 countries [68], persistent disparities in coverage highlight the need for targeted strategies to improve vaccine delivery, financing, and uptake in high-burden regions. Global HPV vaccine coverage trends since 2010 are further illustrated in Figure 2 [69].

## 7. Special Populations

### 7.1. Pregnancy

HPV vaccination is not recommended during pregnancy. However, when the vaccine has been inadvertently administered during pregnancy, safety data have been reassuring [70].

### 7.2. Breastfeeding

According to ACOG guidelines, the HPV vaccine is considered safe during breastfeeding and should be given during this period if the woman is 26 years or younger and has not previously been vaccinated [70]. Shared decision-making should be used for women over 26, as with other adult populations, and should be recommended for high-risk populations.

### 7.3. Adults and Elderly

The Advisory Committee on Immunization Practices (ACIP) recommends HPV vaccination at age 11 or 12, but vaccination can be given starting at age 9. Catch-up vaccination has been recommended in females through 26 years of age since 2006 and for men through 26 years of age since 2019. ACIP does not routinely recommend catch-up HPV vaccination for all adults aged 27 through 45 years but recognizes that some persons who are not adequately vaccinated may be at risk of new HPV infections and might benefit from vaccination. Therefore, ACIP recommends shared clinical decision-making for that age group [71]. The American Cancer Society notably does not endorse vaccination for adults ages 27–45 who are not adequately vaccinated because of low effectiveness and low cancer prevention potential in this age group. Modeling studies estimate that extending vaccination through age 45 years would prevent only an additional 0.5% cases of cancer, 0.4% cases of cervical precancer, and 0.3% cases of genital warts over the next 100 years [72]. Women ages 21–64 years of age should receive cervical cancer screening regardless of their vaccine status, as the vaccine does not protect against all subtypes of HPV. There is no HPV screening test for men [73].

On a global scale, HPV vaccination programs consistently target children, but the upper age limit of HPV vaccination varies by country. In European countries, Moldova and Russia administer the HPV vaccine up to age 45, while Austria administers the vaccine up to age 60, although public funding is only available through age 30 [74,75]. In African countries with limited resources, vaccination efforts are focused on adolescent girls, with WHO aiming to see 90% of girls aged 9–14 vaccinated by 2030 [76]. Similarly, India and other South Asian countries that have launched HPV vaccine programs in the last 5 years have prioritized children and adolescents [77]. Because HPV vaccination is most effective before HPV exposure, global vaccination efforts to this point have primarily focused on the age groups between 9 and 26 in most countries. Consequently, there is a gap in research evaluating the benefits of HPV vaccination in adult and elderly populations at this time.

### 7.4. HIV-Infection/Immunocompromised

A systematic review of HPV prevalence in sub-Saharan Africa found that women living with HIV (WLWH) had a higher prevalence of HPV (53.6%) and of multiple HPV infections (22.6%) compared to HIV-negative women (26.5% and 7.3% respectively) [78]. A systematic review of HPV infection burden in women infected with HIV in LMICs estimated a prevalence of 63% of all HPV subtypes, with 51% prevalence of high-risk HPV strains. The most common HPV subtypes were HPV 16, 18, and 52 [79]. The most recent guidelines from the Infectious Disease Society of America and Office of AIDS Research Advisory Council recommend the nonavalent HPV vaccine as the preferred formulation and a three-dose vaccine schedule for all individuals living with HIV aged 11–26 years. The one dose or two-dose series is not recommended for people with HIV [80]. Since HIV patients are immunocompromised, they are considered a population that is “most likely to benefit” from HPV vaccination through age 45 [81]. Several studies have established the safety and immunogenicity of HPV vaccines in people living with HIV [82,83,84,85]. Immune responses appear stronger among those with suppressed HIV viral loads and higher CD4 counts [84].

## 8. Reasons for Suboptimal Uptake and Strategies to Improve Uptake

Despite longstanding recommendations and demonstrated safety and effectiveness, uptake of the HPV vaccine in the United States remains suboptimal. For the third consecutive year, HPV vaccination coverage has remained unchanged, with 78.2% of adolescents having received at least one dose and only 62.9% being up to date (given previous two-dose recommendation) with the full series [85]. Primary reasons for vaccine hesitancy include perceived lack of provider recommendation, a lack of parental knowledge and necessity especially if an adolescent is not sexually active, safety concerns, and practical access barriers [86,87,88].

### 8.1. Primary Barriers

**Provider-level factors** include inadequate HPV-related knowledge and education amongst health professionals, inconsistent or weak recommendations, tendency to present HPV vaccination as optional rather than routine, and failure to recommend vaccination to younger adolescent patients before sexual debut [88,89,90].

**Parent and patient barriers** reflected common sources of parental hesitancy, including beliefs that the vaccine was not necessary, not recommended, unsafe, parents had limited knowledge or misinformation from the internet, and associations between the HPV vaccine and sexuality [86,91].

**System-level challenges** include logistical barriers (transportation, missed appointments), cost concerns, and lack of reminder systems [88,91].

### 8.2. Evidence-Based Improvement Strategies

**Provider-focused interventions** are essential. A systematic review found that healthcare professionals needed to be better informed and educated on the HPV vaccine. Uptake can be improved by adopting better communication, engagement, supportive information resources, and training for healthcare providers. Healthcare providers must routinely make a strong recommendation to vaccinate patients, both males and females [89]. Training providers to use presumptive language and presenting the HPV vaccine as a vaccine against cancer have been shown to be effective [90].

**Health education interventions** improve uptake. A meta-analysis found that health education improved HPV vaccine uptake compared to usual practice (RR 1.43, 95% CI 1.16 to 1.76; I^2^ = 0%; three studies, 1054 participants; high-certainty evidence) [87]. Information disseminated through handouts and during healthcare visits have been found to be effective at increasing education and vaccine uptake [92]. Culturally sensitive health education can be delivered and tailored by trusted community members including peer school health promotors, religious leaders, and community health workers [93].

**System-level strategies** are effective. A randomized clinical trial found that patient reminder/recall and health-care professional audit/feedback resulted in a statistically significant increase in HPV vaccine uptake compared with usual care [94]. School-based programs have also been effective in increasing vaccine uptake among female students [95]. School-located programs can also address access barriers and reach marginalized populations [92,95].

## 9. Future Directions

Future directions for HPV vaccines include focus on expanding coverage through single-dose vaccine regimens, developing therapeutic vaccines, increasing valency of vaccines, and implementing innovative delivery strategies to achieve global elimination of HPV-related cancers.

Single-dose vaccine regimens have the potential to increase vaccine coverage worldwide and reduce cervical cancer burden and is especially important in resource-limited settings. The WHO Strategic Advisory Group of Experts on Immunization (SAGE) recommended a single-dose schedule in 2022 [96]. As of July 2025, 76 countries had implemented a single-dose schedule [68].

A recent clinical trial published in the New England Journal of Medicine with 20,330 participants found that one dose of either the bivalent or nonavalent HPV vaccine provided protection against HPV 16 and HPV 18 and was not inferior to two doses [97]. Several similar studies in different countries have drawn the same conclusion [98,99,100]. Although long-term immune response of a single-dose HPV vaccination is still needed, which is anticipated from ongoing follow-up of participants in the DoRIS trial in Tanzania and the KEN SHE trial in Kenya, a recent immunobridging analysis [101] of these randomized controlled trials contributes to the increasing evidence challenging the widely accepted thought that protein-based subunit vaccines require a multiple-dose regimen to achieve a long-lasting and adequate immune response.

Given this increasing body of evidence, the CDC has now officially recommended a change to a single-dose HPV vaccine in the US as of January 2026 [102]. This single-dose vaccine regimen could significantly improve supply and demand issues (doubling the number of people vaccinated with the same amount of supply), while decreasing economic cost and logistical barriers to receiving the HPV vaccine, particularly benefiting low- and middle-income countries [103]. An analysis of five LMICs found that cost per adolescent receiving the full schedule could decrease by up to 50% when using a single-dose HPV vaccine regimen instead of a two-dose regimen. A single-dose regimen decreases costs associated with service delivery (via school vaccine programs or travel clinics), costs associated with healthcare worker time, and vaccine procurement and storage costs [104]. Single-dose regimens would also decrease the burden of tracking and following up with patients who need a second dose and save patients time and money associated with multiple healthcare visits [68].

Therapeutic vaccines are a developing area of research. Currently, there is clinical research and development being done using multiple technology platforms including peptides, proteins, DNA, RNA, bacterial vectors, viral vectors, and cell based.

The application of therapeutic HPV vaccines for cervical intraepithelial neoplasia (CIN) focuses mainly on clearing high-risk HPV infections and low-grade lesions, inducing regression of vulvar intraepithelial neoplasia, and inducing regression of CIN2/3. These vaccines have showed promise in phase I-III clinical trials [105].

The application of therapeutic HPV vaccines for recurrent respiratory papillomatosis, caused by HPV types 6 and 11, are also currently being developed. Currently, there are three therapeutic vaccines targeting HPV6 and HPV11 antigens that are in phase I-II clinical trials. One of them, a non-replicating gorilla adenoviral vector-based therapy called Papzimeos (zopapogene imadenovec-drba; PRGN-2012), was approved by the United States FDA in August 2025. Papzimeos encodes HPV6 and HPV11 E6/E7 antigens with the aim to elicit a strong and specific T-cell response that would eliminate HPV6/11-infected cells. Approval was based on a phase I/II clinical trial, in which 51% of patients achieved a complete response to treatment, defined as no need for any RRP surgery for a year following treatment, 86% of patients had a significant reduction in surgical frequency in the first year, 91% in the second year and 95% in the third year [106]. However, this four-dose vaccine over 12 weeks costs $115,000 USD per dose for a total cost of $460,000 USD [107]. The second vaccine is a DNA plasmid vaccine that also encodes for HPV6 and HPV11 E6 and E7 proteins, and the third vaccine is a recombinant Modified Vaccinia Ankara (MVA)-based bovine papillomavirus E2 vaccine. All three vaccines have shown promising results in early-phase clinical trials [106].

High-valent vaccines are currently being developed, as HPV types not covered by vaccines are still at high prevalence [108]. The Gardasil 9 vaccine has the highest number of high-risk HPV serotypes in circulation worldwide but does not fully cover all high-risk HPV types. There are currently higher-valent vaccines in testing, including an 11-valent HPV vaccine developed in China, a 14-valent HPV vaccine called SCT1000 that is in a phase III study, and a 15-valent vaccine developed by Chengda Bio that is currently in development for the prevention of HPV-related diseases [109]. By expanding coverage to additional oncogenic HPV types, higher-valency HPV vaccines would further reduce HPV-related cancers, enhance herd immunity, and further support global prevention efforts.

## 10. Conclusions

The HPV vaccine, first developed over 20 years ago, represents one of the most significant advances in cancer prevention in modern medicine. Extensive clinical trials and real-world data have consistently shown that the HPV vaccine is safe, efficacious and exceptionally effective at decreasing vaccine-type human papillomavirus infection and its sequelae, which range from benign warts to life-threatening cervical, anal, and oropharyngeal cancer. Importantly, the HPV vaccine is most effective when it is administered prior to HPV exposure, underscoring the importance of childhood and early adolescent vaccination.

Despite these successes, HPV vaccination uptake remains low globally. Major disparities in vaccine access and disease burden persist, with low- and middle-income countries bearing the greatest burden of HPV-related cancers. Continued efforts to expand equitable access, reduce costs, implement effective delivery strategies, and ultimately improve coverage are essential to maximize public health impact. At the same time, ongoing research into therapeutic vaccines, higher-valency formulations, and new single-dose vaccine regimens show promise for expanding prevention and treatment options.

Currently, the HPV vaccine has already prevented hundreds of thousands of cancers. By expanding equitable access and uptake worldwide and through continued innovation, the HPV vaccine has the potential to eliminate cervical cancer and substantially reduce the global burden of HPV-related disease.

## Figures and Tables

**Table 1:** Overview of currently licensed prophylactic human papillomavirus (HPV) vaccines.

Vaccine Name	Valency	HPV-Type Coverage	Production System	Licensing
Cervarix	Bivalent	16, 18	Insect Cell-Baculovirus	Licensed in the US by the FDA in 2008
Gardasil	Quadrivalent	6, 11, 16, 18	Saccharomyces cerevisiae (yeast)	Licensed in the US by the FDA in 2006
Gardasil 9	Nonavalent	6, 11, 16, 18, 31, 33, 45, 52, 58	Eukaryotic cells	Licensed in the US by the FDA in 2014
Cecolin	Bivalent	16, 18	Escherichia coli	Licensed in China in 2020; WHO prequalified in 2021
Walrinvax	Bivalent	16, 18	Pichia pastoris (yeast)	Licensed in China in 2022; WHO prequalified in 2022
Cervavac	Quadrivalent	6, 11, 16, 18	Hansenula polymorpha	Licensed in India in 2022
Cecolin 9	Nonavalent	6, 11, 16, 18, 31, 33, 45, 52, 58	Escherichia coli	Licensed in China in 2025

## Data Availability

No new data were created or analyzed in this study.

## Abbreviations

The following abbreviations are used in this manuscript:

HPV	Human papillomavirus
LCR	Long control region
E	Early region
L	Late region
WHO	World Health Organization
VLP	Virus-like particle
HIV	Human immunodeficiency virus
CIN	Cervical intraepithelial neoplasia
AIS	Adenocarcinoma in situ
APC	Annual percentage change
JORRP	Juvenile-onset recurrent respiratory papillomatosis
ICERS	Incremental cost-effectiveness ratios
DALY	Disability-adjusted life year
LMIC	Low- and middle-income countries
HIC	High-income countries
NNV	Number needed to vaccinate
ACOG	American College of Obstetricians and Gynecologists
ACIP	Advisory Committee on Immunization Practices
WLWH	Women living with HIV
SAGE	WHO Strategic Advisory Group of Experts on Immunization
GACVS	Global Advisory Committee on Vaccine Safety
SAE(s)	Serious adverse event(s)
